# Evaluation of Current Community Pharmacist Practice in Saudi Arabia—A Cross-Sectional Study from Pharmacists’ Perspective (Part II)

**DOI:** 10.3390/pharmacy10020038

**Published:** 2022-03-10

**Authors:** Mohammad Alhazmi, Abdulmajeed Bajuayfir, Ejaz Cheema, Mahmoud Elrggal, Majid Ali

**Affiliations:** 1 Pharmacy Services, Ministry of Health, Al-Baha 11176, Saudi Arabia; mohammed.alhazmi9@gmail.com; 2 Retail Department, Al-Dawaa Pharmacies, Al-Khobar 34621, Saudi Arabia; a.bajuayfir@gmail.com; 3 School of Pharmacy, University of Management and Technology, Lahore 54770, Pakistan; ejaz.cheema@umt.edu.pk; 4 Faculty of Pharmacy, Umm Al-Qura University, Makkah 24382, Saudi Arabia; merggal@uqu.edu.sa; 5 School of Life and Medical Sciences, University of Hertfordshire (Hosted by Global Academic Foundation), New Administrative Capital, Cairo 11835, Egypt

**Keywords:** community pharmacy, pharmaceutical care, pharmacist perspective, healthcare service evaluation, Saudi Arabia

## Abstract

This study aimed to evaluate the current practice of community pharmacists from patients’ and pharmacists’ perspectives in Saudi Arabia. This paper presents the pharmacist’s perspective. A cross-sectional self-administered online survey was designed to collect responses from community pharmacists in Saudi Arabia from February to April 2021. The questionnaire consisted of several statements related to best practice in community pharmacy. Pharmacists’ responses to each statement were scored using a 5-point Likert scale. Higher scores represented a greater extent to which they adhered to best practice in the community pharmacy setting and vice versa. Data of 164 participants were included in the analysis. The minimum median score was related to the statement: Pharmacist explains the main side effects. The maximum median score was related to the statement: Pharmacist explains dosage regimen. Pharmacists aged 30 years or above and non-Saudi pharmacists had significantly higher median scores compared with pharmacists less than 30 years of age (*p* = 0.016) and Saudi pharmacists, respectively (*p* = 0.001). A gap between best practice and current practice of community pharmacists was observed. Policymakers can utilize these findings to provide targeted professional development opportunities for the practicing community pharmacists in order to improve the overall service and care for patients.

## 1. Introduction

Professional pharmacy services are defined as “an action or set of actions undertaken in or organized by a pharmacy, delivered by a pharmacist or other health practitioner, who applies their specialized health knowledge personally or via an intermediary, with a patient/client, population or other healthcare professionals, to optimize the process of care, with the aim to improve health outcomes, and the value of healthcare” [1]. Community pharmacies are considered the first in line for patients regarding ease of access to obtain their medication and health services. Pharmacists in community pharmacies can provide medical services for numerous patients in a day with or without appointments. These services can be seeking advice, reassurance, treatment, or even a combination of all these [2].

The core functions of the community pharmacist range from recommending suitable non-prescription products to detecting and minimizing any side effects that may harm patients related to the prescription medicines, among which patient counseling is an integral part [3]. Thus, there are many factors that contribute to the satisfaction of the patient. A significant increase in the number of patients worldwide who visit community pharmacies as compared to health care centers can be attributed to several reasons, including the low cost of services offered by the pharmacies, less waiting time, and more time spent with the pharmacist. Saudi Arabia is no different. Ample evidence suggests that people in Saudi Arabia frequently visit their community pharmacies for various reasons [4,5,6,7,8]. Hence, there is an increased public demand for utilizing community pharmacy services. There is also an opportunity for community pharmacies to contribute to the country’s Vision 2030, which promises public–private partnerships for effective primary health care [9,10]. Because of these reasons, it is imperative to provide good-quality and standardized community pharmacy services. This study aimed to evaluate the current practice of community pharmacists from patients’ and pharmacists’ perspectives in Saudi Arabia in order to identify the potential areas for improvement that can ultimately be used to develop recommendations for policymakers. This paper presents the methods, results, discussion, and conclusions related to the pharmacist’s perspective. The patient perspective is presented in Part I of our study.

## 2. Methods

### 2.1. Study Design

A cross-sectional self-administered online survey was employed in this study to collect the pharmacists’ responses.

### 2.2. Instrument

We developed the survey instrument (questionnaire) based on the relevant literature and personal observations and practices [11,12,13,14,15]. The questionnaire consisted of three parts. Part I comprised the demographic characteristics of the pharmacists, such as gender, age, the geographical location where they were practicing, nationality, and educational background. Part II consisted of questions related to their working experience in Saudi Arabia and knowledge of any regulations regarding pharmacist and community pharmacist practice in Saudi Arabia. Part III comprised statements related to best practice in community pharmacy. A 5-point Likert scale was used for each statement to record pharmacists’ responses. Pharmacists’ responses to each statement were scored to assess the extent to which they perform best practice in community pharmacies (Never = 1, Rarely = 2, Sometimes = 3, Often = 4, Always = 5). Higher scores represented a greater extent to which they adhere to best practice in the community pharmacy setting and vice versa.

In order to detect pharmacists who randomly selected options on the Likert scale, we included the following statement in the questionnaire with Likert scale options: *Ask for extra money from the patient.* As opposed to the other statements, the ‘always’ option for this statement would illustrate worst practice by the pharmacist. It was decided to delete all the data from the analysis for these pharmacists, who may have selected ‘always’ for all the statements (including this one), assuming that this would indicate random responses to the statements by the pharmacist.

The questionnaire was developed and administered in the English language. The questionnaire was piloted with three pharmacists, and minor changes were made in the wording of some questions.

### 2.3. Validity and Reliability of the Instrument

We ensured the face validity of the questionnaire with the help of two experienced community pharmacists and two expert academic researchers to establish the relevance and reasonability of the questions without any ambiguity. They also checked the questionnaire’s content to ensure that the instrument was logical and easy to understand (content validity). The reliability analysis of Part III of the instrument showed a Cronbach’s alpha value of 0.91, indicating strong internal consistency.

### 2.4. Participants and Setting

All pharmacists working in the community pharmacy setting in Saudi Arabia were eligible to participate in the survey. The survey was disseminated via an online link using Google Forms on various social media accounts and relevant WhatsApp groups for pharmacists, and no incentives or compensation was offered for completing the survey. The data were collected from February 2021 to April 2021.

### 2.5. Statistical Analysis

The data were downloaded from Google Forms as an Excel file and then exported to SPSS (Version 24) for descriptive and inferential statistical analyses. Only the researchers had access to the files. The descriptive analysis demonstrated patients’ demographic characteristics and responses in frequencies, percentages, and medians with standard deviations. Furthermore, the Mann–Whitney U *t*-test and Kruskal–Wallis test were utilized to determine the effect of the independent variables (gender, age, geographical location, nationality, and marital status) on the dependent variables (median score of all the statements), and a *p*-value of less than 0.05 was considered statistically significant.

Currently, there are an estimated 24,395 community pharmacists dispensing pharmaceutical products in community pharmacies across the country [16]. The sample size determined by an online sample size calculator (SurveyMonkey, San Mateo, CA, USA) with a 95% confidence interval and a 5% margin error was 379 community pharmacists.

### 2.6. Ethical Considerations

This study was reviewed and approved by the Institutional Review Board (IRB) of Umm Al-Qura University (approval number: HAPO-02-K-012-2021-04-655). The survey introduction informed the pharmacists about their voluntary participation in the survey, the anonymity and confidentiality of the collected data, and their right to withdraw their information at any time by contacting the researchers.

## 3. Results

### 3.1. Demographics

A total of 169 pharmacists responded to the survey. The data of five participants were deleted because they were deemed random responses (explanation in the methods section). The data of 164 participants were included in the analysis. More than half of them were males (*n* = 116; 71%). The majority of the participants were Saudi nationals (*n* = 122; 75%) and less than 30 years old (*n* = 123; 75%). The northern region of Saudi Arabia was the geographical location where the highest percentage of participants responded from (*n* = 64; 39%). Table 1 shows more details regarding the demographic characteristics of the participants.

### 3.2. Responses to the Statements

Participants’ responses to the statements are illustrated in Table 2. The median score of the individual statements ranged from 3.26 (2.00) to 4.34 (1.01) (5 being the maximum possible score for each statement). The minimum median score was related to the statement: *Pharmacist explains the main side effects*. The maximum median score was related to the statement: *Pharmacist explains dosage regimen (dose and frequency).* The overall median score of all the statements was 77 (14), ranging from 30 to 100 (maximum possible score of 100).

The Mann–Whitney U *t*-test revealed no significant difference in the median scores between male and female pharmacists: 3.86 (0.67) versus 3.81 (0.75); *p* = 0.958. However, pharmacists aged 30 years or above and non-Saudi pharmacists had significantly higher median scores compared with pharmacists less than 30 years of age (*p* = 0.016) and Saudi pharmacists (*p* = 0.001), respectively. Moreover, those who were aware of any regulation about pharmacists’ role in Saudi Arabia and aware of any regulation about community pharmacists’ role in Saudi Arabia also had significantly higher median scores compared with those who were not aware.

The Kruskal–Wallis test revealed a statistically significant difference in the median scores of the participants from different regions (*p* = 0.008). Participants from the northern region had the highest median score, 4.03 (0.64), whereas participants from the central region had the lowest median score, 3.44 (0.78). No significant difference was observed in the median scores of the pharmacists with different educational backgrounds and different working experiences in Saudi Arabia.

## 4. Discussion

Our study was an exploratory study in which we evaluated the current practice of community pharmacists from their own perspective in Saudi Arabia. In previous studies, community pharmacists have successfully been able to self-evaluate their practices and competencies [17,18]. Standards for the quality of pharmacy services have been set by the World Health Organization [11]. Studies from other countries have shown varying results related to the evaluation of community pharmacy services [13,14,15]. In our study, a scale based on best community pharmacist practices was developed utilizing the literature, and community pharmacists were asked to rate their practice on this scale, following which we quantified their responses [11,12,13,14,15].

We found that the median score of male and female pharmacists was above 3.8, and there was no significant difference found between them, meaning they evaluated themselves to be performing best practice equally well. However, the number of male pharmacists in our study sample was more than double that of female pharmacists. This reflects the effect of culture on the workforce in Saudi Arabia, in which females are still reluctant to accept retail jobs. However, because of the recent reforms taking place in the country, a shift in the workforce is anticipated [19,20,21]. Alhaddad and colleagues evaluated the satisfaction of female patients with the services received from male community pharmacists in Saudi Arabia and reported that less than half of patients were satisfied with the services they received from male pharmacists; more than half were embarrassed to discuss sensitive female issues with the male pharmacists and preferred the presence of female pharmacists in community pharmacies [22]. With the anticipated shift in the workforce in the country, we expect to have more female pharmacists available in community pharmacies to serve female patients.

We also found that we had a higher number of young and newly graduated Saudi pharmacists in our study sample. This again reflects the workforce reforms taking place in Saudi Arabia, which is creating a significantly increased number of job opportunities for young people in every sector compared with past numbers. With more young pharmacists joining the workforce, we can expect an improvement in the quality of pharmaceutical care provided in community pharmacies; one study reported that younger pharmacists are generally more supportive of pharmaceutical care and exhibit best practice of pharmaceutical care [23]. Another promising finding was that majority of the pharmacists were aware of the pharmacy regulations in the country. However, it was alarming to observe that approximately 20% of the pharmacists were not aware of any pharmacy regulations. This raises serious concerns regarding the qualification and licensing of these pharmacists to practice in community pharmacies.

Our study also revealed a significant difference in the median scores of community pharmacists from different regions of the country. The community pharmacists in the northern region had the highest score, whereas those in the central region had the lowest score, and this difference was significant. This coincides with the patient evaluation of community pharmacist practices reported in Part I of our study, in which patients in the northern region also scored significantly higher compared with patients in the central region. It must be noted that the population demographics of Saudi Arabia vary from region to region [24]. Some areas are more populated with non-Saudis, and others are more populated with the people of middle age and the elderly. Moreover, the central region is the most populous, and community pharmacists may not have sufficient time to provide good-quality pharmacy services to patients in busy community pharmacies [25]. Furthermore, in community pharmacies, patients prefer a private area for discussing personal health issues [26], and this may not be possible in crowded community pharmacies. By contrast, the northern region is less populous than the central region, and pharmacists may have ample time to build good relationships and rapport with their patients in community pharmacies. This may explain the reasons for the differences in patient expectations and community pharmacist practices in these regions.

The statement on which the pharmacists scored the lowest compared with the other statements was: “Explain main side effects (to the patients)”. This coincides with findings of the patient evaluation of community pharmacist practices reported in Part I of our study, in which this was the statement that was scored lowest by the patients. It also resonates with other studies in which community pharmacists in Saudi Arabia have been reported to be reluctant to explain medication side effects to their patients [4,25]. This may be because business still plays a crucial role in the community pharmacy practice, and the pharmacists may not be willing to explain medication side effects to patients lest this turns them away from community pharmacies.

Other statements on which both community pharmacists and patients (according to findings on the patient perspective reported in Part I of our study) scored low were: “Discuss complementary medications the patient may be taking”, “Discuss general health issues of the patient”, and “Follow up with the patients about their health”. Al-Arifi also reported that community pharmacists in Saudi Arabia did not provide adequate counseling to patients regarding complementary medications [27]. Likewise, Khdour and Hallaki reported that pharmacists generally do not discuss general health issues with patients in community pharmacies [28].

The four statements that achieved a median score of more than 4 (out of total 5) were: “Explain dosage regimen (dose and frequency) to the patient”, “Explain dosage regimen (administration) to the patient”, “Ensure understanding of dosage regimen by the patient”, and “Use simple (non-medical) terms when talking to the patient or customer”. Interestingly, the first two statements and the last statement coincided with the statements that were also scored higher than 4 by the patients when evaluating the community pharmacy practice (reported in Part I of our study). A study from the United Arab Emirates reported that community pharmacists explain to patients how to use their medication on 98% of occasions [29]. By contrast, in a study from Iraq, community pharmacists were not generally found to check the prescriptions for dose and frequency accuracy [30].

### Limitations

The findings of our study must be interpreted considering the limitations. It was only an exploratory study, and we were not able to achieve the required sample size because of time constraints. Moreover, we utilized a self-completed online questionnaire that could be subject to the misunderstanding of the questions and memory bias.

## 5. Conclusions

Despite the limited sample size, our results provide insight into the status of current practice by community pharmacists in Saudi Arabia from their own perspective. Our study highlights a gap between current practice and best practice of community pharmacists as evaluated by the pharmacists. The pharmacists should consider including a clear and elaborate explanation of the main medication side effects; counseling on complementary medications the patient might be taking or be interested in taking; and discussing general health issues related to their interactions with the patients in community pharmacies. They should also follow up with the patients about their medications and health whenever possible. Policymakers can utilize these findings to provide targeted professional development opportunities for practicing community pharmacists in order to improve the overall service and care for patients.

## Figures and Tables

**Table 1:** Demographic characteristics of the participants (*n* = 164) with the comparison of their median scores.

Demographic Characteristics	Number of Participants	Median Score (IQR)	*p*-Value
**Gender**			0.958
Male	116	3.86 (0.67)	
Female	48	3.81 (0.75)	
**Age**			0.017
Less than 30 years	123	3.77 (0.68)	
30 years or above	41	4.06 (0.69)	
**Geographical location**			0.008
Central region	22	3.44 (0.78)	
Eastern region	14	3.68 (0.58)	
Northern region	64	4.03 (0.64)	
Southern region	28	3.79 (0.73)	
Western region	36	3.87 (0.65)	
**Nationality**			0.003
Saudi	122	3.75 (0.70)	
Non-Saudi	42	4.12 (0.58)	
**Educational background**			0.431
BPharm	85	3.92 (0.63)	
PharmD	70	3.76 (0.70)	
Postgraduate degree	9	3.75 (1.14)	
**Working experience as a pharmacist in KSA**			0.265
1 year	83	3.80 (0.59)	
2 years	25	3.81 (0.92)	
3 or more years	56	3.93 (0.73)	
**Aware of any regulation about pharmacist role in Saudi Arabia**			<0.001
Yes	130	3.93 (0.68)	
No	34	3.50 (0.64)	
**Aware of any regulation about community pharmacist role in Saudi Arabia**			0.018
Yes	110	3.94 (0.67)	
No	54	3.66 (0.70)	

IQR: interquartile range.

**Table 2:** Participants’ frequencies (with percentages) and the median scores (with standard deviation) for the statements.

Statement	Never (%)	Rarely (%)	Sometimes (%)	Often (%)	Always (%)	Median (IQR)
1. Ask about current medications of the patient	11 (6.7)	10 (6.1)	38 (23.2)	40 (24.4)	65 (39.6)	3.84 (1.21)
2. Ask about comorbid diseases the patient may have	8 (4.9)	12 (7.3)	36 (22)	52 (31.7)	56 (34.1)	3.83 (1.13)
3. Explain dosage regimen (dose and frequency) to the patient	8 (4.9)	5 (3)	17 (10.4)	28 (17.1)	106 (64.6)	4.34 (1.10)
4. Explain dosage regimen (administration) to the patient	10 (6.1)	3 (1.8)	16 (9.8)	30 (18.3)	105 (64)	4.32 (1.12)
5. Ensure understanding of dosage regimen by the patient	8 (4.9)	6 (3.7)	20 (12.2)	33 (20.1)	97 (59.1)	4.25 (1.12)
6. Explain main side effects	12 (7.3)	31 (18.9)	57 (34.8)	30 (18.3)	34 (20.7)	3.26 (1.20)
7. Ask for extra money from the patient *	132 (80.5)	7 (4.3)	10 (6.1)	5 (3.0)	10 (6.1)	1.5 (1.1)
8. Discuss complementary medications the patient may be taking	8 (4.9)	18 (11)	58 (35.4)	33 (20.1)	47 (28.7)	3.57 (1.16)
9. Discuss general health issues of the patient	5 (3)	16 (9.8)	57 (34.8)	37 (22.6)	49 (29.9)	3.66 (1.10)
10. Discuss the patient’s concerns about medications	6 (3.7)	14 (8.5)	55 (33.5)	42 (25.6)	47 (28.7)	3.67 (1.09)
11. Use simple (non-medical) terms when talking to the patient or customer	0 (0)	8 (4.9)	38 (23.2)	30 (18.3)	88 (53.7)	4.21 (0.96)
12. Ensure patient compliance with the medications	8 (4.9)	10 (6.1)	49 (29.9)	31 (18.9)	66 (40.2)	3.84 (1.17)
13. Provide the patient written information about medications whenever needed	6 (3.7)	14 (8.5)	31 (18.9)	38 (23.2)	75 (45.7)	3.99 (1.15)
14. Handle consultations on sensitive topics appropriately	8 (4.9)	13 (7.9)	44 (26.8)	37 (22.6)	62 (37.8)	3.8 (1.17)
15. Provide clear non-verbal instructions on patient counseling for patients/customers who cannot hear	11 (6.7)	13 (7.9)	37 (22.6)	40 (24.4)	63 (38.4)	3.8 (1.22)
16. Follow up with the patients about their health	12 (7.3)	28 (17.1)	50 (30.5)	32 (19.5)	42 (25.6)	3.39 (1.24)
17. Solve medication-related problems whenever needed	4 (2.4)	21 (12.8)	49 (29.9)	39 (23.8)	51 (31.1)	3.68 (1.12)
18. Promote health awareness	4 (2.4)	11 (6.7)	41 (25)	37 (22.6)	71 (43.3)	3.98 (1.09)
19. Provide patient care relevant to the Saudi culture	11 (6.7)	8 (4.9)	43 (26.2)	36 (22)	66 (40.2)	3.84 (1.20)
20. Follow the mechanisms for checking dispensing procedures in the pharmacy	10 (6.1)	18 (11)	30 (18.3)	40 (24.4)	66 (40.2)	3.82 (1.25)
21. Ensure proper labeling of the medications before dispensing	5 (3)	16 (9.8)	36 (22)	40 (24.4)	67 (40.9)	3.9 (1.14)

IQR: interquartile range. * Not included in the analysis.

## Data Availability

The data presented in this study are available upon request from the corresponding author.
